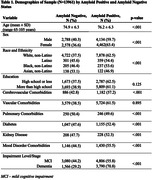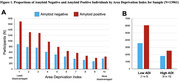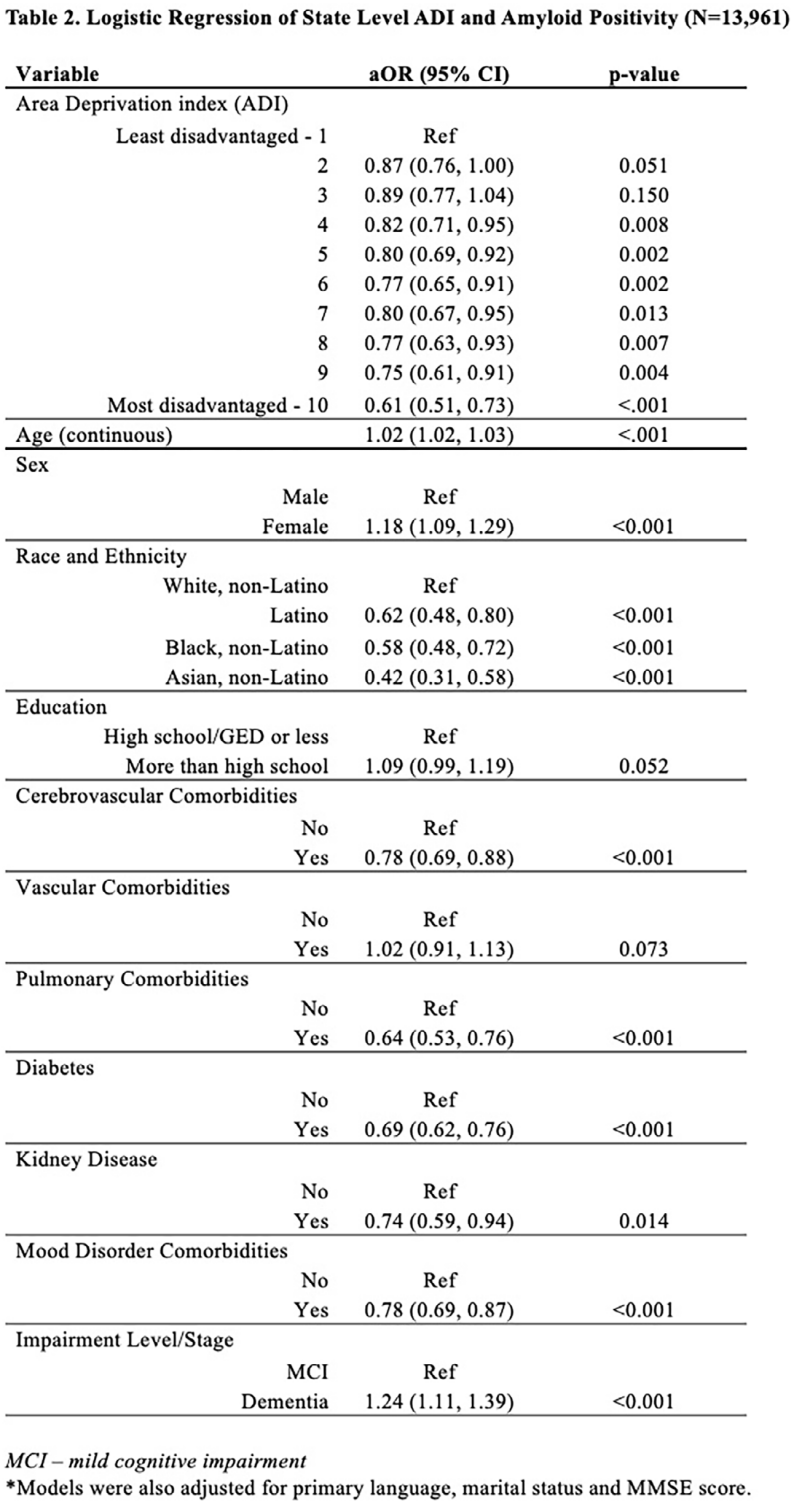# Residential Neighborhood Disadvantage and Amyloid Positivity: Findings from IDEAS

**DOI:** 10.1002/alz.091995

**Published:** 2025-01-09

**Authors:** Charles Windon, Elena Tsoy, Jennifer Livaudais‐Toman, Lucy Hanna, Constantine Gatsonis, Maria C. Carrillo, Charles Apgar, Peggye Dilworth‐Anderson, Leah Karliner, Bruce E Hillner, Torsten B Neilands, Nynikka Palmer, Katherine L Possin, Anita L. Stewart, Rachel A. Whitmer, Consuelo H. Wilkins, Gil D. Rabinovici

**Affiliations:** ^1^ Memory and Aging Center, Weill Institute for Neurosciences, University of California, San Francisco, San Francisco, CA USA; ^2^ University of California, San Francisco, San Francisco, CA USA; ^3^ Brown University, Providence, RI USA; ^4^ Alzheimer's Association, Chicago, IL USA; ^5^ American College of Radiology, Reston, VA USA; ^6^ University of North Carolina, Chapel Hill, Chapel Hill, NC USA; ^7^ University of California San Francisco, San Francisco, CA USA; ^8^ Virginia Commonwealth University, Richmond, VA USA; ^9^ UCSF, San Francisco, CA USA; ^10^ University of California, Davis School of Medicine, Sacramento, CA USA; ^11^ Vanderbilt University Medical Center, Nashville, TN USA

## Abstract

**Background:**

Residence in a disadvantaged neighborhood (e.g., high poverty rate, poor housing, etc.) is associated with greater dementia risk and possibly greater postmortem Alzheimer’s pathology. It remains unknown if neighborhood disadvantage is associated with in vivo beta‐amyloid positron emission tomography (PET) for Alzheimer’s. We examined this using data from the Imaging Dementia Evidence for Amyloid Scanning (IDEAS) study.

**Methods:**

IDEAS captured >18,000 PET scans among cognitively impaired Medicare beneficiaries from 595 US dementia clinics between 2016‐2018. We defined neighborhood disadvantage using Area Deprivation Index (ADI), a validated composite of 17 social determinants measures captured in American Community Survey and US Census data. IDEAS participant zip code data was linked to census block group for ADI value calculation through geocoding.

Association between visual interpretation of PET in IDEAS (positive/negative) and state‐level ADI (1‐10; 10 being greatest disadvantage) was examined via logistic regression controlling for covariates (age, sex, education, level of impairment (MCI vs dementia), race/ethnicity, comorbid conditions) with cluster adjusted standard errors by practice location.

**Results:**

Among 13,961 cognitively impaired participants, 61.6% were amyloid positive, 56.5% had MCI, and 50.4% were female (Table 1). The majority (90.2%) were non‐Latino White with 4.7% Latino, 3.2% Black, and 1.9% Asian representation (Table 1). Participants were highly educated (68.1% beyond high school) and 31.0% (4,333/13,961) resided in neighborhoods of greater disadvantage (ADI ≥ 6) (Figure 1).

Greater ADI was associated with lower odds of amyloid positivity (aOR 0.61, 95% CI 0.51‐0.73, p<.001 for ADI 10 vs ADI 2 and aOR 0.87, 0.76‐1.00, p .051 for ADI 2 vs. ADI 1(ref)). Latino (aOR 0.62, 0.48‐0.80, p<.001), Black (aOR 0.58, 0.48‐0.72, p<.001), and Asian (aOR 0.42, 0.31‐0.58, p<.001) identity was associated with lower odds of amyloid positivity along with multiple comorbidities (Table 2).

**Conclusion:**

We observed a relationship between residence in a more disadvantaged neighborhood (higher ADI) and lower rates of amyloid positivity. Given this finding and known association between neighborhood disadvantage and postmortem AD pathology, it is possible non‐amyloid pathology also contributes towards cognitive impairment among individuals from disadvantaged neighborhoods and among diverse groups. This has implications for clinical use of novel amyloid lowering therapies.